# Hybrid carbon thermal interface materials for thermoelectric generator devices

**DOI:** 10.1038/s41598-020-75976-9

**Published:** 2020-11-02

**Authors:** Seok-Hwan Chung, Jong Tae Kim, Dong Hwan Kim

**Affiliations:** Materials Research Institute, Daegu Gyeongbuk Institute of Science and Technology (DGIST), Daegu, 42988 South Korea

**Keywords:** Carbon nanotubes and fullerenes, Electronic devices

## Abstract

Thermal interface materials (TIMs) are extensively used in electronic devices as efficient heat transfer materials. We fabricated all-carbon TIMs by hybridizing single-wall carbon nanotubes (SWCNTs) with graphite and demonstrated their performance by applying them to a thermoelectric generator (TEG) device. The hybrid carbon TIM exhibited maximum thermal conductivity when the SWCNT content was near 10 wt%. The TIM thermal contact resistance measured by a home-made calorimeter setup was 2.19 × 10^−4 ^m^2^K/W, which did not vary with temperature but decreased with applied pressure. Post-treatment of the TIM with a silane coupling agent further reduced the TIM thermal contact resistance by 30%. When the TIM was placed between a TEG device and a copper heat reservoir, the TEG output power increased with the temperature difference across the TEG and applied pressure. Moreover, the post-treatment of the TIM enhanced the output power of the TEG device by up to 18.5%. This work provides a simple and effective pathway towards a carbon-based TIM that can be applied to a high temperature TEG.

## Introduction

Continuing progress in device miniaturization in the electronics industry requires a more effective method to manage excess heat so as to protect the device functionality. A thermal interface material (TIM) is a functional material inserted between a heat source and a heat sink to improve heat transfer efficiency^1–3^. TIMs play an important role in dissipating the excess heat generated by electronic devices, enhancing their efficiency and lifetime. Conventional TIMs are fabricated by dispersing metal or metal oxide fillers with high thermal conductivity in a polymer matrix. To achieve efficient heat transfer, TIMs need to have a continuous heat-conducting network with high thermal conductivity, low interface thermal resistance, high thermal/mechanical stability and satisfactory wetting properties at heterogeneous interfaces.

Recently, many studies have reported TIMs composed of nano- or micron-scale carbon such as carbon nanotubes (CNT)^4–8^, graphene^9–11^ and graphite^12–15^. Carbon-based materials have a very high intrinsic thermal conductivity due to strong *sp*^2^ bonding. These materials also have the advantages of low thermal expansion, mechanical strength, flexibility and low weight. The addition of small amounts of CNT filler is known to significantly increase the thermal conductivity of composite TIMs. TIMs with CNTs are fabricated by either dispersing CNT fillers in a polymer matrix^5^ or by growing CNTs on a substrate^6–8^. However, fabricating an efficient TIM with a CNT filler is difficult because of the low filling fraction, low degree of dispersion and high thermal interface resistance of CNTs^16,17^. To reduce the high thermal resistance at the interface of CNT–CNT or CNT-contact materials, various approaches have been recently reported, such as carbon-based bonding^18,19^, metal-based bonding^20–24^, infiltration with phase change materials (PCMs) or epoxy^7,25^, polymer coating^26^ and chemical functionalization^27–29^.

The main applications of TIMs have been in the fields of microelectronics, power electronics and LED lighting^1,3,30,31^. Although less explored, another important field of TIM application is the thermoelectric generator (TEG). The TEG converts wasted heat energy into useful electric energy through the Seebeck effect^32,33^. It is considered as a heat engine working between a hot and a cold heat reservoir. The temperature of the heat source ranges from body heat up to more than 300 °C for automotive and industrial applications^33^. To maximize the output power of a TEG system, not only a high-performance TEG device is required, but also stable and efficient thermal contacts. Effective TIMs can reduce the thermal contact resistance between a TEG and the heat reservoirs by increasing the contact area and filling the air gap between the interfaces. Since TIMs play an important role in obtaining the maximum possible power out of a TEG module by maintaining a large temperature difference across the TEG, the study of the application of TIMs to a TEG system has gained recent interest^34–36^. However, the number of studies on this topic is still limited.

In this work, we fabricated all-carbon TIMs by hybridizing single-wall carbon nanotubes (SWCNTs) with graphite and investigated their thermal transport properties using a laser flash method and a home-made calorimeter setup. The optimal TIM composition for the maximum thermal conductivity was determined. The thermal contact resistance of the TIM was characterized as a function of the temperature and applied pressure across the TIM. The TIM was also evaluated through the output performance of a TEG device with a TIM placed between the TEG device and a copper (Cu) block. In addition, we demonstrated a post-treatment method that improved the performance of the TEG device by further reducing the TIM thermal contact resistance.

## Results and discussion

**Figure 1:**
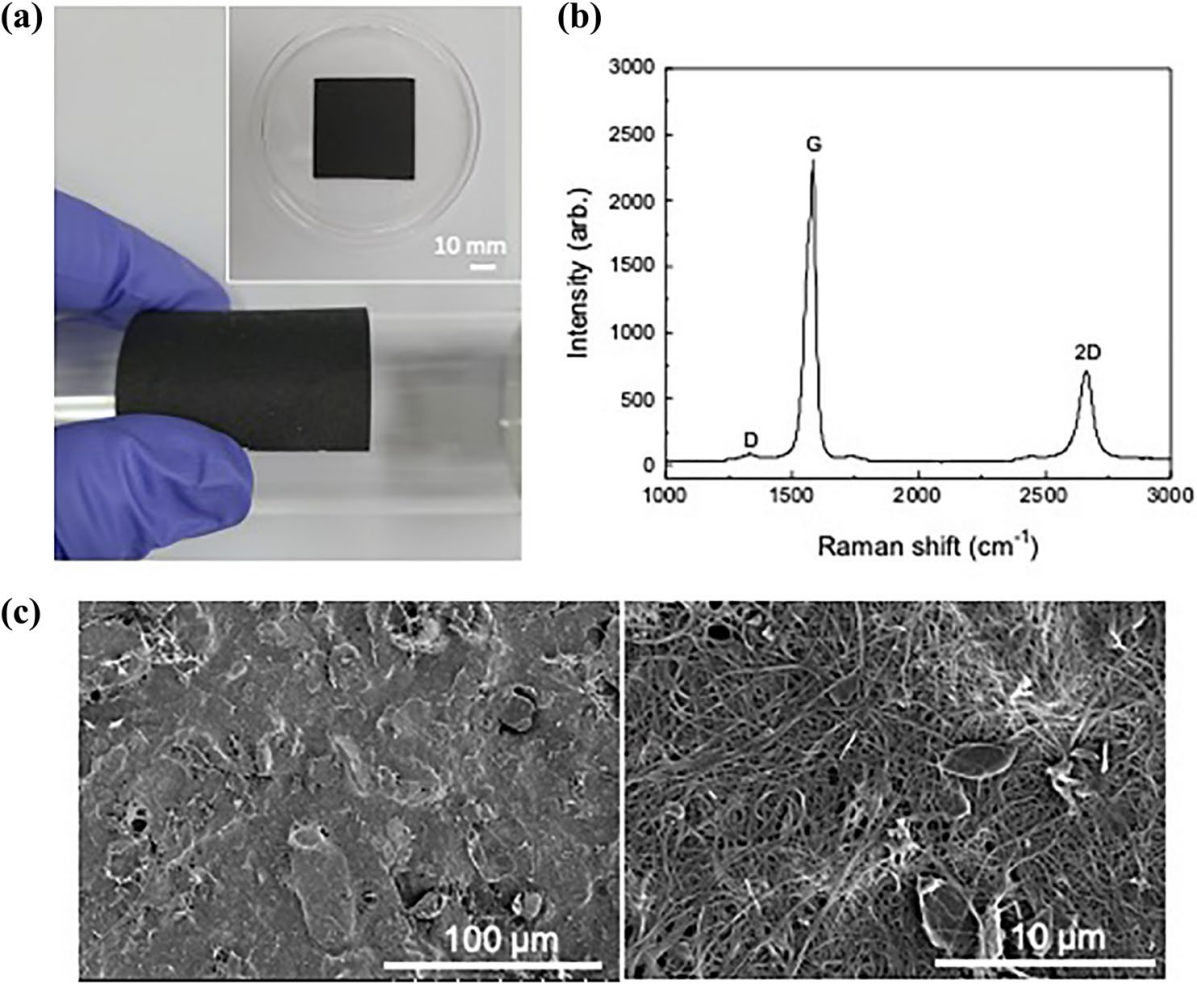
(**a**) Photograph of a hybrid carbon TIM (*f* = 12 wt%). (**b**) Raman spectrum and (**c**) surface FE-SEM images of the TIM at low (left) and high (right) resolution.

Figure 1 shows the photograph, Raman spectra and surface FE-SEM images of a hybrid carbon TIM with SWCNT weight fraction *f* = 12 wt%. The fabricated TIM in Fig. 1a is a bendable and compressible dark gray sheet. The Raman spectrum of the TIM in Fig. 1b shows peaks at 1335, 1583 and 2663 cm^−1^ that correspond to the D, G and 2D peaks of SWCNT and graphite^37^. The surface FE-SEM images in Fig. 1c clearly show that the SWCNTs bridge and cross-link the large rigid graphite platelets with line contacts. The flexibility of the TIM originates from that of SWCNTs with high aspect ratios (2500–30,000). The mechanical flexibility is important for utilizing nanoscale carbon as a solid-state gap-filling material. Furthermore, compared to using a single filler in TIM, hybrid fillers in TIM have advantages such as additional channels of heat flow with improved inter-filler contact and mechanical strength by connecting the neighboring fillers^38^. Figure 1c shows that the majority of the cross-linking SWCNTs and graphite platelets are oriented parallel to the TIM surface. This observation agrees with the results in previous literature, which reported that rigid micro-particles with high aspect ratios have a strong tendency to align with a substrate^39^. The SWCNTs and graphite platelets were aligned with the surface of the filter membrane during the vacuum filtering process. The X-ray diffraction pattern of the TIM (Fig. S1, supplementary data) also indicates the in-plane orientation of the graphite platelets, since the (002) and (004) peaks associated with the (*a,b*) plane of the graphite are dominant, while other peaks are suppressed.

### Thermal conductivity of the hybrid carbon TIM

**Figure 2:**
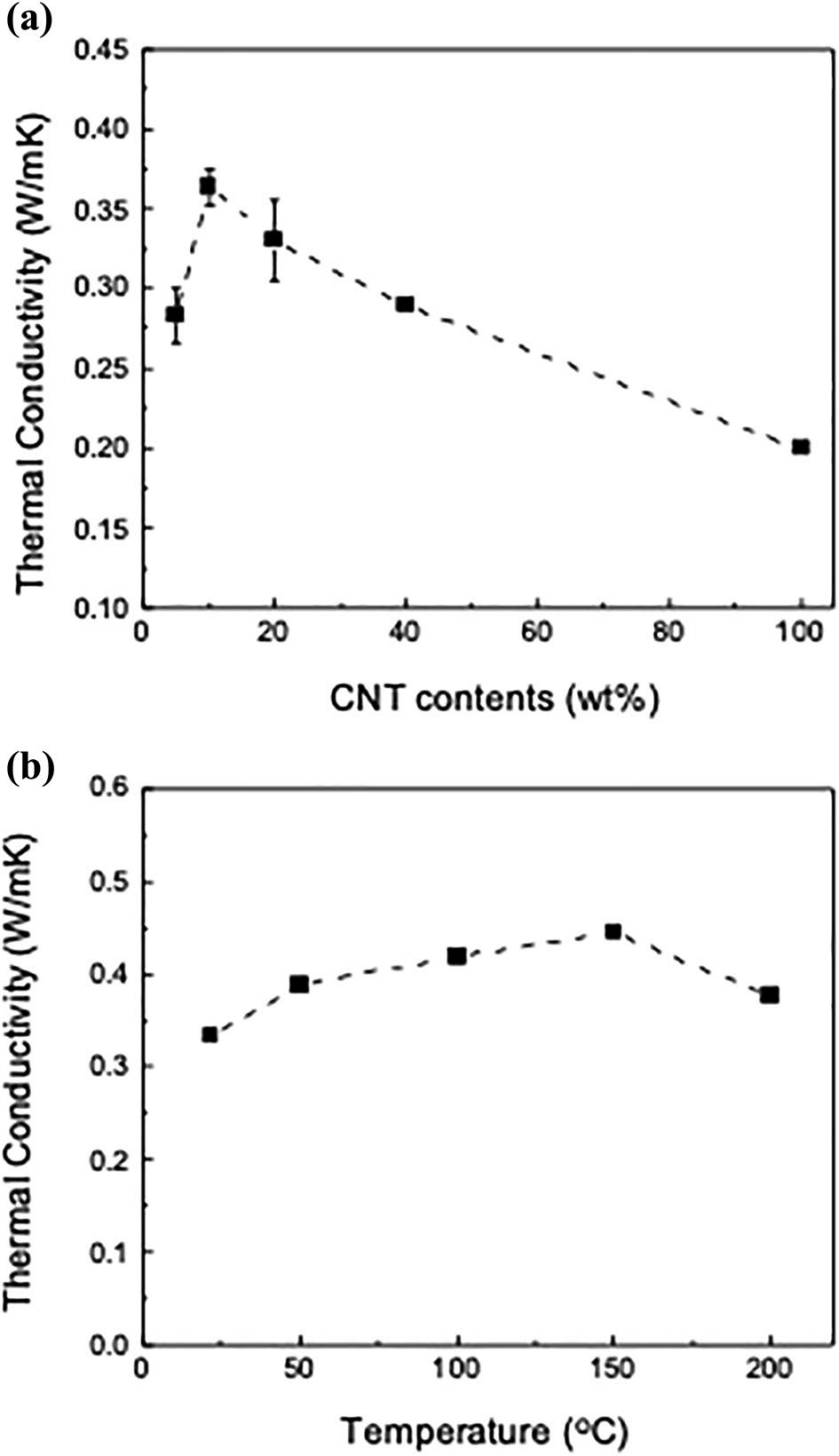
(**a**) Thermal conductivity of TIMs with different SWCNT contents (*f* = 5, 10, 20, 40 and 100 wt%). (**b**) Temperature dependence of thermal conductivity of a TIM (*f* = 10 wt%).

Figure 2a shows the thermal conductivity of the TIMs with *f* = 5, 10, 20, 40 and 100 wt%. The thermal conductivity was calculated using the relationship $\kappa$(*T*) = *ρ*(*T*) × *C*_*p*_(*T*) × *α*(*T*), where *ρ* is the mass density of the sample, *C*_*p*_ is the specific heat capacity, and *α* is the thermal diffusivity measured by the laser flash method^40–42^. The thermal conductivity of the TIM in the through-plane direction is low, owing to the in-plane alignment and the large anisotropy in the thermal conductivity of the SWCNTs and graphite^43,44^. The thermal conductivity increases from 0.28 W/mK for *f* = 5 wt% to 0.36 W/mK for *f* = 10 wt%, and then decreases to 0.20 W/mK for *f* = 100 wt%. This result contradicts the general rule of mixtures that predicts a monotonous decrease in the thermal conductivity of composites^38^. Therefore, the peak at *f* = 10 wt% indicates that the most synergistic effect of SWCNT-graphite hybridization occurs near the weight fraction due to enhanced heat transfer through the linear contacts between the SWCNT and graphite. Figure 2b shows the temperature dependence of the thermal conductivity of the TIM with *f* = 10 wt%. The thermal conductivity is in the range of 0.33 to 0.44 W/mK. It slightly increases up to 150 °C first, due to the increase in specific heat capacity *C*_*p*_ with temperature, and then slightly decreases at 200 °C, due to the decrease in the thermal diffusivity *α* at high temperature.

### Thermal contact resistance of the hybrid carbon TIM

The thermal contact resistance of the TIMs was obtained by a home-made calorimeter setup. The measurement principle is based on one-dimensional steady-state heat conduction^7,36^. In this work, the thermal contact resistance ${\rho }_{c}$ is defined by.

1 $${\rho }_{c}={R}_{T}A=\left({R}_{TIM}+{R}_{i1}+{R}_{i2}\right)A=\frac{t}{{\kappa }_{TIM}}+{R}_{i1}A+{R}_{i2}A,$$

where $A$ is the contact area, and ${R}_{TIM}{, R}_{i1}$ and ${R}_{i2}$ are the thermal resistances of the TIM, interface 1 and interface 2. *t* is the thickness and ${\kappa }_{TIM}$ is the thermal conductivity of the TIM. However, the determination of ${\rho }_{c}$ by direct measurement of ${R}_{i1}$ and ${R}_{i2}$ is technically challenging. For this reason, we obtained ${\rho }_{c}$ using the following.

2 $${\rho }_{c}={R}_{T}A= \frac{A}{{Q}_{A}} ({T}_{H}-{T}_{C}),$$

where ${Q}_{A}$ is the average heat flow and ${T}_{H}$ and ${T}_{C}$ are the temperature values of the hot and cold surfaces of the TIM, respectively. The thermal contact resistance of the TIM strongly depends on not only the thermal conductivity of the TIM but also the physical properties of the interface, such as the contact pressure and surface roughness.

**Figure 3:**
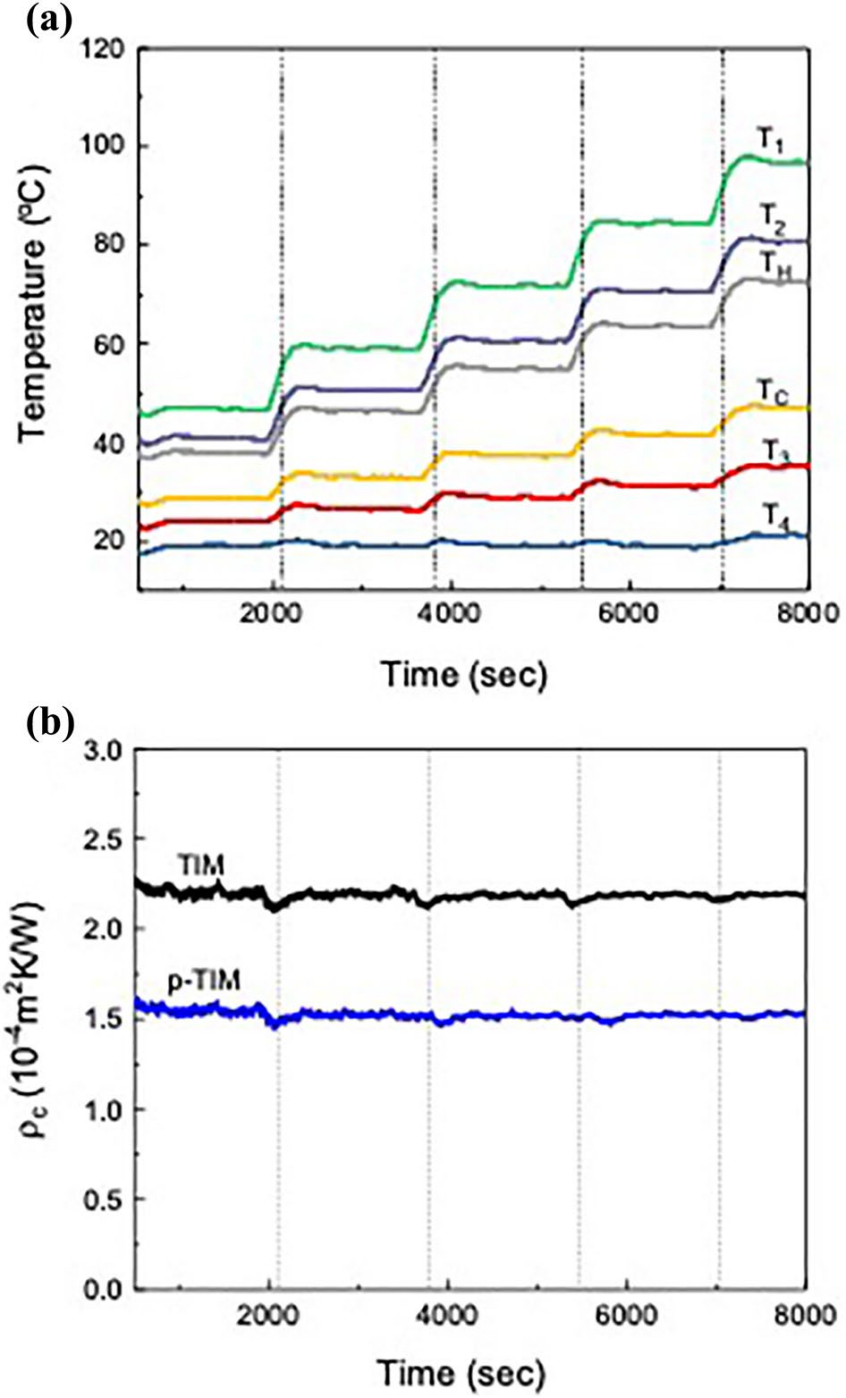
(**a**) Temperature changes at *T*_1_,* T*_2_,* T*_*H*_,* T*_*C*_,* T*_3_ and *T*_4_ with a TIM (*f* = 10 wt%, 95 μm thick) when the hot side temperature *T*_*2*_ was raised from 40 to 80 °C with 10 °C step, while the cold side temperature *T*_*4*_ was fixed at 20 °C. The applied pressure was 0.37 MPa. (**b**) Thermal contact resistance of a TIM and a TIM post-treated with 1 wt% silane solution (p-TIM).

Figure 3a shows the temperature changes at *T*_1_, *T*_2_, *T*_*H*_, *T*_*C*_, *T*_3_ and *T*_4_ of the calorimeter with a TIM (*f* = 10 wt%, 95 μm thick). The hot side temperature *T*_2_ was increased from 40 to 80 °C with a step of 10 °C, while the cold side temperature *T*_4_ was fixed at 20 °C. The applied pressure was 0.37 MPa. The temperature changes stepwise and the transition time between each setpoint is approximately 5 min. In Fig. 3b, the thermal contact resistance of the TIM was calculated from the measurement data using Eq. (2). Regardless of the temperature increase, ${\rho }_{c}$ has a constant value of 2.19 × 10^–4^ m^2^K/W since both the average heat flow ${Q}_{A}$ and the temperature difference across the TIM, *T*_*H*_*–T*_*C*_, increase proportionally. In general, the thermal contact resistance decreases with increasing interface temperature due to either the lower tensile strength of the contact materials or the higher thermal conductivity of the air within the micro-gap at the interface^45^. However, in the low-temperature range, ${\rho }_{c}$ does not change with increasing temperature due to the thermal stability of the TIM and the negligible changes in heat transfer through the air gap. When the TIM was post-treated with 1 wt% silane solution (p-TIM), the thermal contact resistance became 1.52 × 10^–4 ^m^2^K/W (Fig. 3b), which is approximately 30% lower than that of the un-treated TIM. This result indicates that the post-treatment improved the TIM performance by forming better interfacial contact. Silane has been known as a coupling agent for surface hydroxyl groups^46–48^ and was used to enhance thermal transport through the CNT array and graphene^28,29^. The chemical modification of the TIM surface was confirmed using an energy dispersion X-ray (EDX) analyzer and a Fourier transform infrared (FTIR) spectrometer (Figs. S2 and S3 (a), supplementary data).

**Figure 4:**
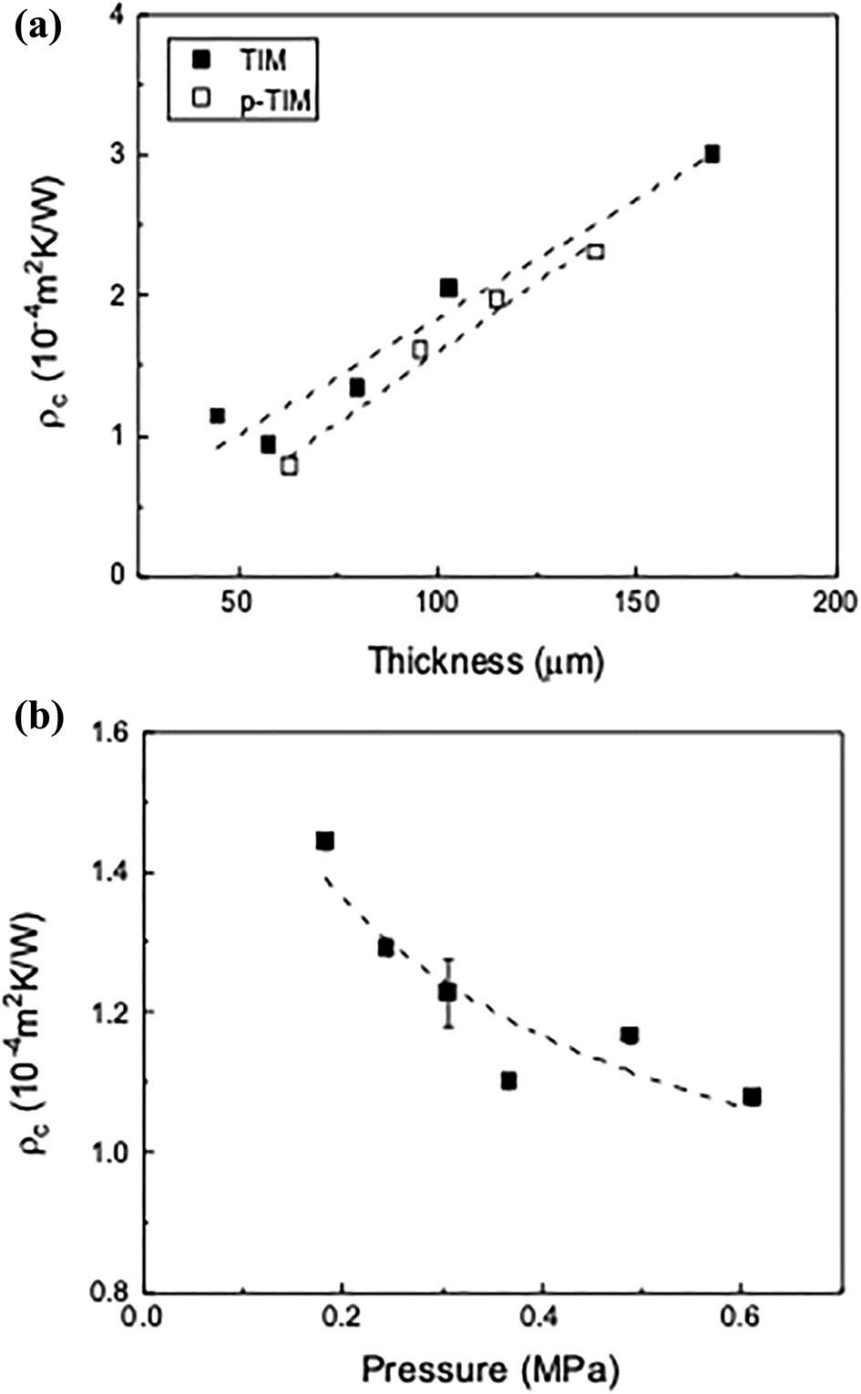
(**a**) Thermal contact resistance of TIMs and p-TIMs (*f* = 10 wt%) versus sample thickness. The applied pressure was 0.37 MPa. (**b**) Thermal contact resistance of a p-TIM (*f* = 10 wt%) versus applied pressure. The hot side temperature was 70 °C and the cold side temperature was 20 °C.

The thickness dependence of the thermal contact resistance of the TIM (*f* = 10 wt%) is shown in Fig. 4a. The applied pressure was 0.37 MPa. The thermal contact resistance ${\rho }_{c}$ has a linear dependence on the thickness, increasing from 1.1 to 3.0 × 10^–4 ^m^2^K/W as the thickness increases from 45 to 169 μm. We note that, in previous studies, the thermal contact resistance between a Cu plate and vertically grown CNTs on a Si substrate under similar compressive force was 0.2 to 0.4 × 10^–4 ^m^2^K/W due to lower film thickness and better interface compatibility^7,20^. The TIM thermal conductivity can be obtained from the slope of the linear fit of the measured data to Eq. (1). The calculated TIM thermal conductivity was 0.61 W/mK. Compared to the thermal conductivity obtained by the laser flash method in Fig. 2, the calculated thermal conductivity is higher due to the compressive force exerted during the measurement. The thermal conductivity of the p-TIM is 0.50 W/mK, which is lower than that of the untreated TIM because the uncoupled silane under compression may block the thermal contact between the carbons^49^. However, since the total thermal contact resistance of the p-TIM is reduced, this result indicates that the p-TIM has lower interfacial thermal resistance due to better interface wetting properties.

Figure 4b shows the pressure dependence of the thermal contact resistance of a p-TIM (*f* = 10 wt%) with an initial thickness of 85 μm. The hot and cold side temperature values were fixed at 70 and 20 °C, respectively. The thermal contact resistance exhibits a non-linear decrease with the applied pressure. As the pressure increases from 0.18 to 0.61 MPa, the thermal contact resistance decreases from 1.44 to 1.08 × 10^–4 ^m^2^K/W. Its pressure dependence is known to vary with the composition, coverage and mechanical property of the contact^50,51^. When the flexible TIM was compressed by a pressure applied in the normal direction, the surface of the TIM conformed well to the rigid surface of the AlN plates. The TIM thermal contact resistance decreases due to (1) the decrease in the interface thermal resistance with the better conformability at higher load, and (2) the increase in the thermal conductivity for increasing density. The SWCNT-graphite network in the TIM improved the thermal contact by filling the microscopic air gaps originating from the roughness of the AlN plates. The RMS roughness of the AlN plate was 0.7 μm. On the other hand, the RMS roughness of the TIM was 1.6 μm before compression, decreasing to 1.0 μm after compression. The TIM thickness after applying pressure was 64 μm, which corresponds to 25% of the compressibility. The TIM surface morphology before and after applying 0.61 MPa of normal pressure is shown in Fig. S4 of the supplementary data.

### Application of the hybrid carbon TIM to a TEG device

**Figure 5:**
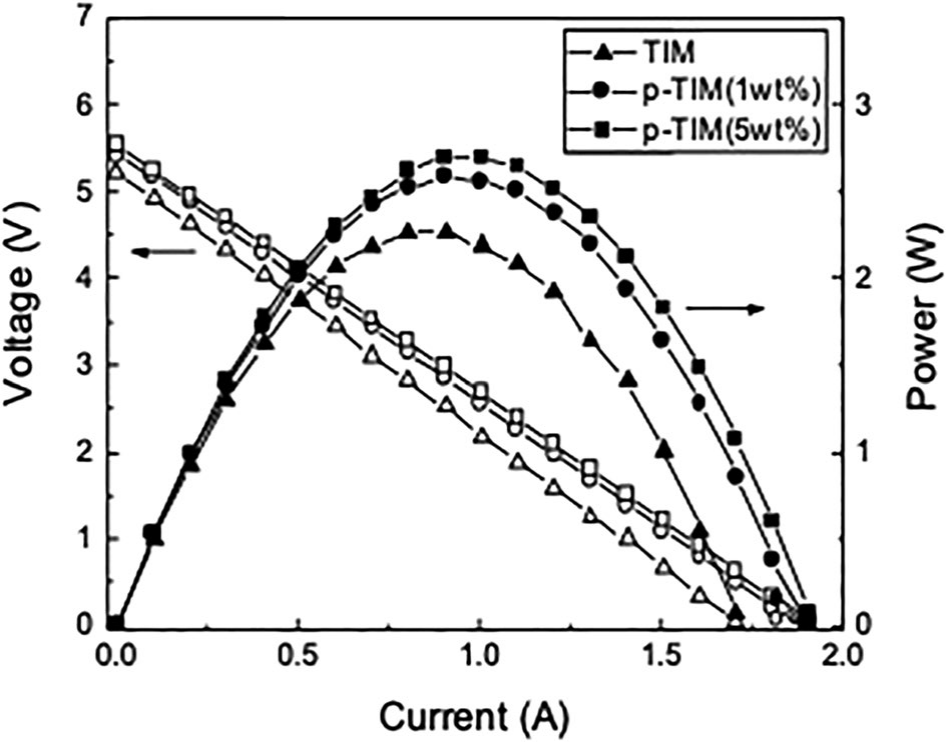
Voltage and power versus current when a TIM (*f* = 10 wt%) or p-TIMs treated with 1 wt% and 5 wt% silane solution is applied at the cold side of a TEG. The temperature difference and the pressure were 130 °C and 0.37 MPa, respectively.

We applied TIMs to a TEG device and evaluated them through the output performance of the device. Figure 5 shows the current dependence of the TEG voltage and power when a TIM (*f* = 10 wt%), a p-TIM treated with 1 wt% silane solution, or a p-TIM treated with 5 wt% silane solution was applied at the cold side of the TEG. The temperature difference $\Delta$*T* was 130 °C and the pressure was 0.37 MPa. The *I–V* plots are linear with constant slopes. The *x*- and *y*-intercepts, defined as short-circuit current *I*_*sc*_ and open-circuit voltage *V*_*oc*_, increase with the concentration of the silane coupling agent. Since the *I–V* plots are linear, the output power *P* is a quadratic function of the current. The maximum output power *P*_*max*_ of the TEG is 2.27 W before the post-treatment of the TIM. However, after post-treatment with 1 wt% and 5 wt% solution of the silane coupling agent, *P*_*max*_ increases to 2.58 W and 2.69 W, which correspond to 13.7% and 18.5% improvement, respectively. The chemical functionalization of the TIM enhanced the heat transfer between the TEG and the heat reservoir by improving the interface wetting property, which resulted in the enhancement of the temperature difference across the TEG (${\Delta T}_{TEG})$. The FTIR spectrum (Fig. S3(b), supplementary data) suggests that the silane coupling agent can form covalent bonding to the Cu heat reservoir, which may reduce the thermal contact resistance by suppressing phonon scattering at the interface.

**Figure 6:**
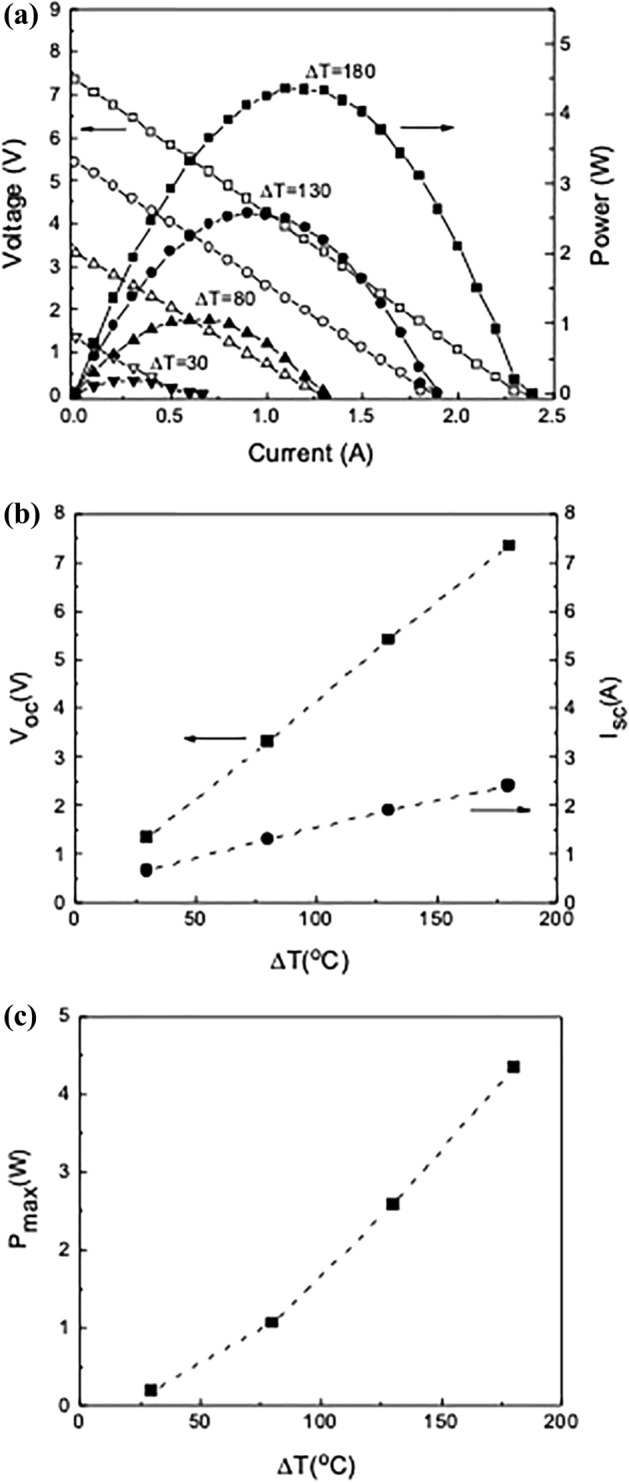
(**a**) Voltage and power versus current at temperature difference $\Delta {T}$ of 30, 80, 130 and 180 °C when a p-TIM (*f* = 10 wt%) is applied at the cold side of a TEG. The applied pressure was 0.37 MPa. (**b**) Open-circuit voltage *V*_*oc*_ and short-circuit current *I*_*sc*_ versus $\Delta$ *T.* (**c**) Maximum power *P*_*max*_ versus $\Delta$*T.*

Figure 6 shows the temperature dependence of the TEG voltage and power when a p-TIM is applied at the cold side of the TEG device. The temperature difference $\Delta T={T}_{2}-{T}_{3}$ was 30, 80, 130 and 180 °C, while the pressure was 0.37 MPa. As shown in Fig. 6a, *I*_*sc*_ and *V*_*oc*_ increase as $\Delta T$ increases from 30 to 180 °C. The slope of the *I–V* plots $|\Delta V/\Delta I|$ representing the source resistance increases from 2.1 to 3.1 $\Omega$ as $\Delta T$ increases. The maximum output power ${P}_{max}={I}_{sc}{V}_{oc}/4$ also increases with $\Delta T$ up to 4.35 W at $\Delta T$ = 180 °C. *I*_*sc*_ and *V*_*oc*_ increase linearly with $\Delta T$, as shown in Fig. 6b. The effective Seebeck coefficient *S*_*eff*_ of the TEG is defined by ${S}_{eff}=\Delta V/{\Delta T}_{TEG}$, where ${\Delta T}_{TEG}=\gamma \Delta T \left(\gamma <1\right)$^52^. Since $\gamma$ = 0.87 with the p-TIM at the cold side, *S*_*eff*_ of the TEG device is 47.8 mV/K. Figure 6c shows the quadratic dependence of *P*_*max*_ on $\Delta T$, which is due to the linear dependence of both *I*_*sc*_ and *V*_*oc*_ on $\Delta T$. Efficient TIM enables large TEG output power by maintaining a large temperature difference across the TEG (${\Delta T}_{TEG}$).

**Figure 7:**
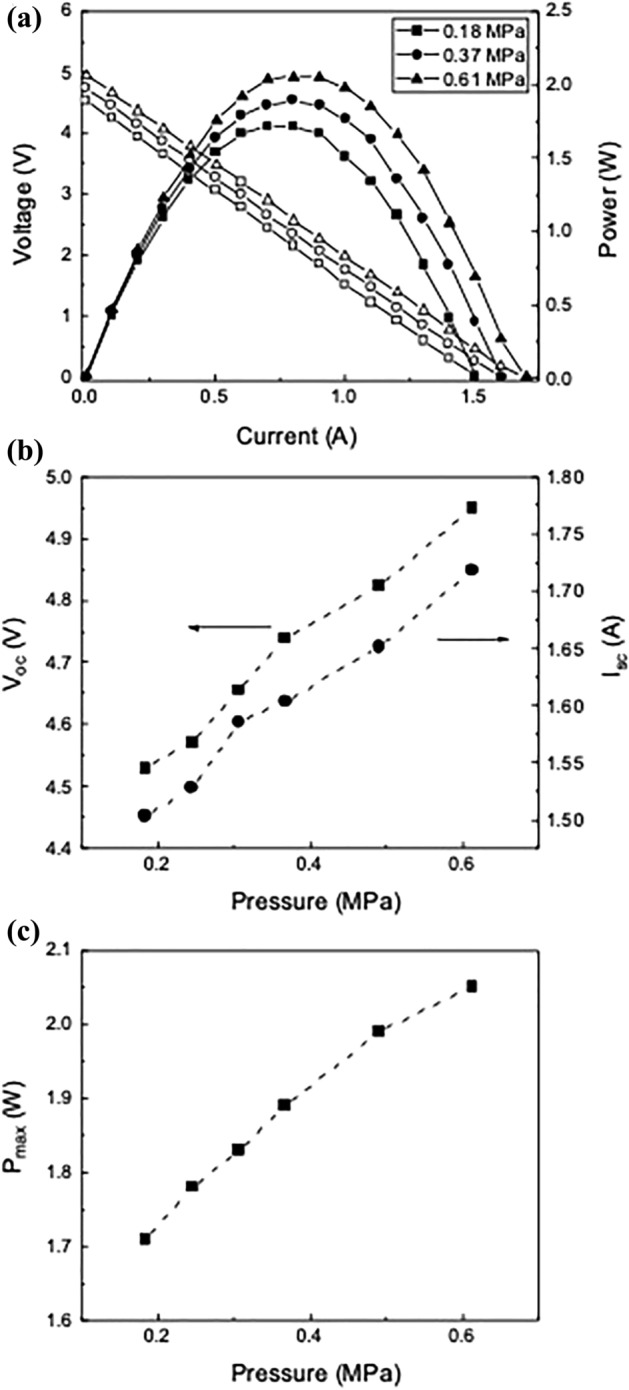
(**a**) Voltage and power versus current at different applied pressure when two p-TIMs (*f* = 10 wt%) are applied at the hot and the cold side of a TEG. The temperature difference $\Delta {T}$ is 130 °C. (**b**) Open-circuit voltage *V*_*oc*_ and maximum power *P*_*max*_ versus applied pressure. (**c**) Maximum power *P*_*max*_ versus applied pressure.

When a TIM is applied between a TEG device and a heat reservoir, normal pressure improves the thermal conductance at the interface by reducing the thermal contact resistance of the TIM. Figure 7a shows the output voltage and power versus current at different values of pressure when two p-TIMs (*f* = 10 wt%) are applied at both the hot and cold sides of the TEG device. The temperature difference $\Delta T={T}_{2}-{T}_{3}$ was fixed at 130 °C. When the pressure increases from 0.18 to 0.61 MPa, *P*_*max*_ increases from 1.71 to 2.05 W (20% increase). *I*_*sc*_, *V*_*oc*_ and *P*_*max*_ also increase as pressure increases, as shown in Fig. 7b,c. Assuming that the heat loss is negligible, the temperature difference $\Delta T$ can be expressed as

3 $$\Delta T={Q}_{A}\times ({R}_{Cu}+{R}_{TIM1}+{R}_{TEG}{ + R}_{TIM2})$$

where ${Q}_{A}$ is the average heat flow and ${R}_{Cu}$,${R}_{TIM1}$,${R}_{TEG}$, and ${R}_{TIM2}$ are the thermal resistances of Cu, TIM 1, TEG and TIM 2. The reduced thermal contact resistance of TIM 1 and TIM 2 observed when pressure is applied, causes an increase in the TEG output power due to increased heat flow through the TEG device. The thermal resistance consists of those of the micro-contact and micro-gap at the interface, both of which decrease with applied pressure^50,51^. Since the rate of change of the thermal resistance decreases as pressure increases (Fig. 4b), the output power gain of the TEG slightly decreases at high pressure as shown in Fig. 7c.

To achieve the optimal performance of a TEG, the temperature difference between the hot and cold sides of the TEG must be maximized by maximizing the thermal conductance at the interfaces. Currently, thermal grease (TG) is widely used as a heat-transporting material in electronic devices. TG is usually composed of metal oxide particle fillers in silicone oil. However, since the TG is liquid-based, it is difficult to handle and requires uniform application across the interface to ensure a good thermal conductance. In addition, the operation is usually limited to a low temperature range (< 200 °C)^53^. Therefore, the TG is not suitable for long-term operation of TEG devices in contact with high temperature heat sources. The hybrid carbon TIM, on the other hand, is more robust, easy to handle and can be operated in a high temperature range. Further optimization of the microstructure, mechanical properties and interface chemistry, especially perpendicular alignment of CNT and graphite, will improve the performance of carbon-based TIMs for a wide range of applications to high-temperature TEGs.

## Conclusion

In this work, we fabricated all-carbon TIMs by hybridizing SWCNTs with graphite, and studied their thermal transport properties using a laser flash method and a home-made calorimeter setup. The hybridization of SWCNTs with graphite showed the most synergistic effect on thermal conductivity when the SWCNT content was near 10 wt%. The thermal contact resistance of the TIM placed between two AlN plates was 2.19 × 10^–4 ^m^2^K/W. The TIM thermal contact resistance did not depend on the temperature difference across the TIM but decreased with the applied pressure. The output power of a TEG device with a TIM placed between the device and Cu heat reservoirs increased with the temperature difference and applied pressure. Post-treatment of the TIM with a silane coupling agent further reduced the thermal contact resistance of the TIM by 30% and improved the output power of the TEG device by up to 18.5%. In the future, we expect to improve the performance of the hybrid carbon TIMs by further optimizing the microstructure, mechanical properties and interface chemistry.

## Methods

### Preparation of the hybrid carbon TIM

High-purity SWCNT powder (> 98%, 1–2 nm in diameter, 5–30 μm in length, Avention) and synthetic graphite powder (99%, 7–11 μm, Alfa Aesar) were magnetically stirred in a 3:1 solution (100 mL) of H_2_SO_4_ and HNO_3_ for 12 h and 24 h, respectively. They were dried in an oven at 80 °C after vacuum filtering and washing with de-ionized (DI) water. Subsequently, the SWCNT powder (15–90 mg) and graphite powder (150–900 mg) were mixed and dispersed in anhydrous ethanol (30–60 mL) by ultrasonic treatment (300 W) for 3 h. The weight fraction *f* of the SWCNT powder to the graphite powder was selected to be between 5 and 40 wt%. The acid treatment removed catalyst particles and improved the dispersion of the SWCNTs and graphite by hydroxylic or carboxylic surface functionalization. The chemical modification is also known to reduce thermal boundary resistance at the carbon–carbon interfaces in TIM^54,55^. After ultrasonic treatment, the mixture was slowly vacuum filtered using a microporous glass membrane filter. Next, the SWCNT-graphite composite on the membrane filter was annealed at 80 °C for 5 min. Finally, the hybrid carbon TIM was obtained after delaminating the sheet from the membrane filter. The size of the all-carbon TIM was 40 × 40 mm^2^, and the thickness was between 50 and 250 μm. For the post-treatment, the TIM sheet was treated with 20 mL of 1–5 wt% (3-mercaptopropyl)trimethoxysilane (> 95%, Sigma-Aldrich) in ethanol for 3 h at 60 °C for silanization. After the reaction, the TIM was washed with DI water and dried at 100 °C for 10 min.

### Characterization of the TIM

The surface morphology of the TIMs was observed using field-emission scanning electron microscopy (FE-SEM, SU8230, Hitachi). Their Raman spectra were observed using a Raman spectroscopy system (Nicolet Almega XR, Thermo Scientific) with a laser wavelength of 532 nm. The surface roughness was characterized by a profilometer (Alpha-Step, KLA Tencor).

### Thermal conductivity measurement

The thermal diffusivity of the TIMs with a square area of 8 × 8 mm^2^ was measured by a laser flash method (LFA457, Netzsch)^40–42^. The thermal conductivity was evaluated using the relationship $\kappa$(*T*) = *ρ*(*T*) × *C*_*p*_(*T*) × *α*(*T*), where *ρ* is the mass density of the sample, *C*_*p*_ is the specific heat capacity, and *α* is the thermal diffusivity. The mass density was measured by Archimedes’ principle (XT 220A, Precisa) and the specific heat capacity was measured using a differential scanning calorimeter (DSC, DSC 200 F3, Netzsch).

### Thermal contact resistance measurement

**Figure 8:**
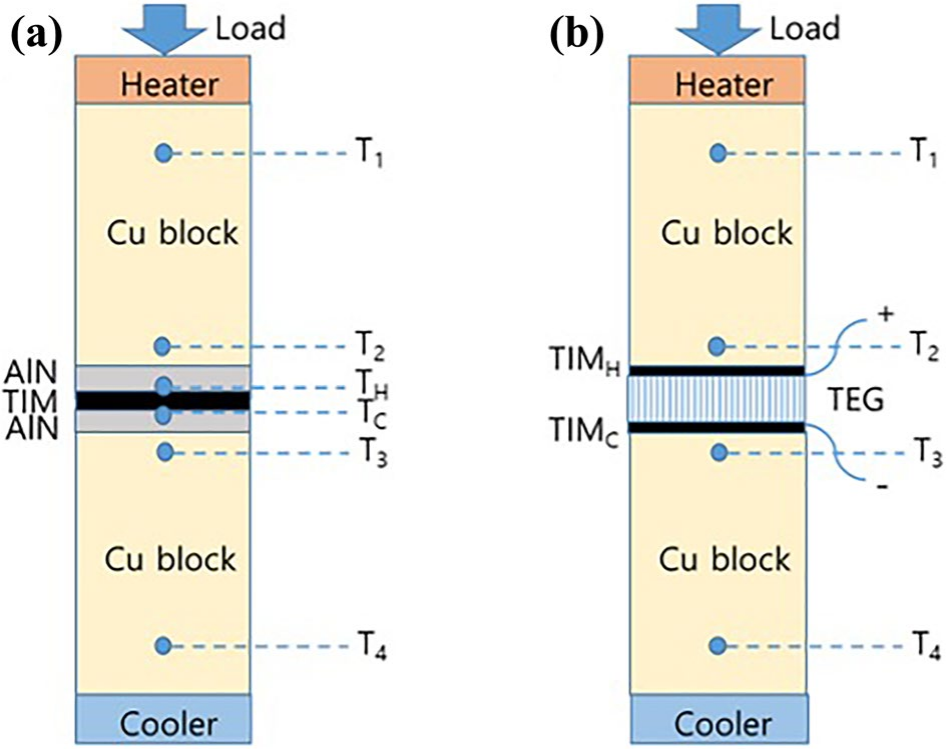
Schematics of the experimental configuration for (**a**) the measurement of the thermal contact resistance of a TIM and (**b**) the evaluation of TIM applied to a TEG device.

A home-made calorimeter setup was used to measure the thermal contact resistance of the TIMs (Fig. 8a). A Cu block with an electrical heater was used as the hot heat reservoir and another Cu block with cooling water from a chiller was employed as the cold heat reservoir. The cross-sectional area of the Cu blocks was 40 × 40 mm^2^. The temperature values at the designated locations of each Cu block (*T*_1_, *T*_2_, *T*_3_ and *T*_4_) were measured using K-type thermocouples. *T*_2_ and *T*_3_ were used to control the temperature of the hot and cold Cu blocks, respectively. A controlled pressure between 0.18 and 0.61 MPa was exerted from the top Cu block by a load cell. For the measurement of the TIM thermal contact resistance, a TIM sheet was applied between two aluminum nitride (AlN) plates attached to the Cu blocks, as shown in Fig. 8a. K-type thermocouples were embedded at the surface of the AlN plates to directly measure the temperature difference *T*_*H*_*–T*_*C*_ between the top and bottom interfaces with the TIM.

### Evaluation of the TIM applied to a TEG device

For the evaluation of the TIM performance, the TIM was applied at the top or bottom interface between a commercial TEG (Jeongkwan Materials) and the Cu blocks, as shown in Fig. 8b. The generated voltage and power of the TEG as a function of current were recorded under a given temperature difference and pressure when thermal equilibrium was established. The measurements were performed under a low vacuum condition to prevent convective heat loss.

## Supplementary information

Supplementary Information
