# Supplementary material for: Hybrid carbon thermal interface materials for thermoelectric generator devices
Source: Sci Rep. 2020 Nov 2;10:18854. doi: 10.1038/s41598-020-75976-9 (PMC7606529; doi:10.1038/s41598-020-75976-9)
Supplement: Supplementary file 1 — Supplementary Information [file 41598_2020_75976_MOESM1_ESM.pdf]

Supplementary Information

Hybrid carbon thermal interface materials for thermoelectric generator devices

Seok-Hwan Chung*, Jong Tae Kim, Dong Hwan Kim

Materials Research Institute, Daegu Gyeongbuk Institute of Science and Technology (DGIST), Daegu 42988, South Korea

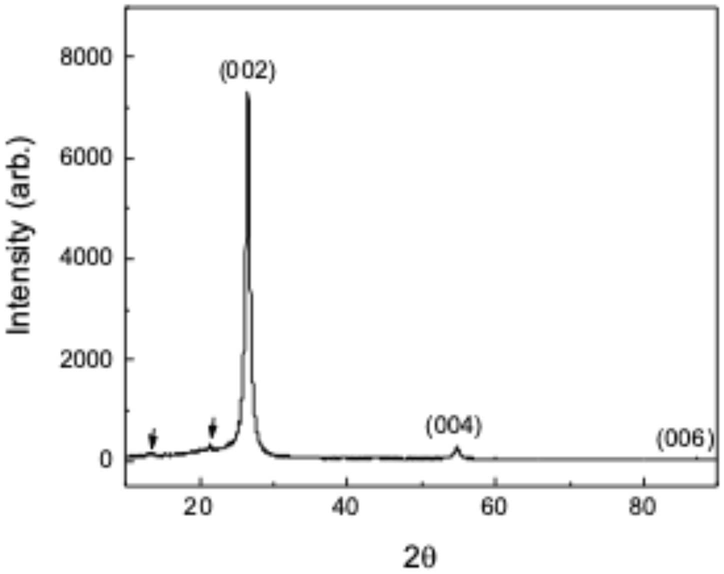

Figure S1 X-ray diffraction (XRD) pattern of a hybrid carbon TIM (f = 12 wt%) measured by an XRD spectrometer (MiniFlex II, Rigaku) using Cu Kα radiation (30 kV, 15 mA). Two peaks indicated by the arrows are associated with the formation of graphite oxide.

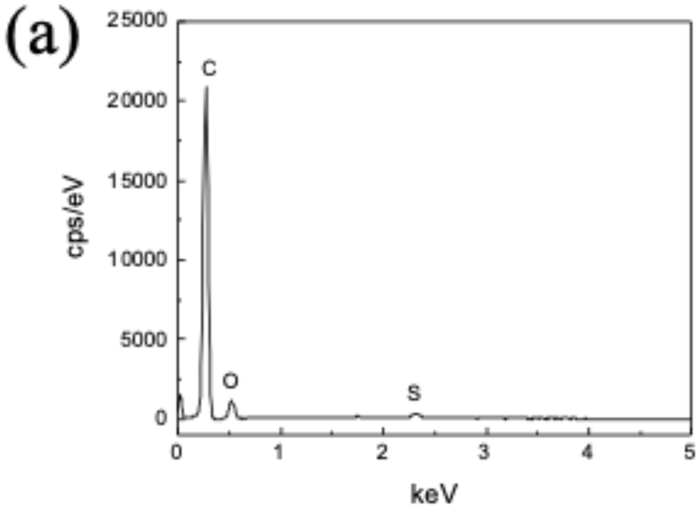

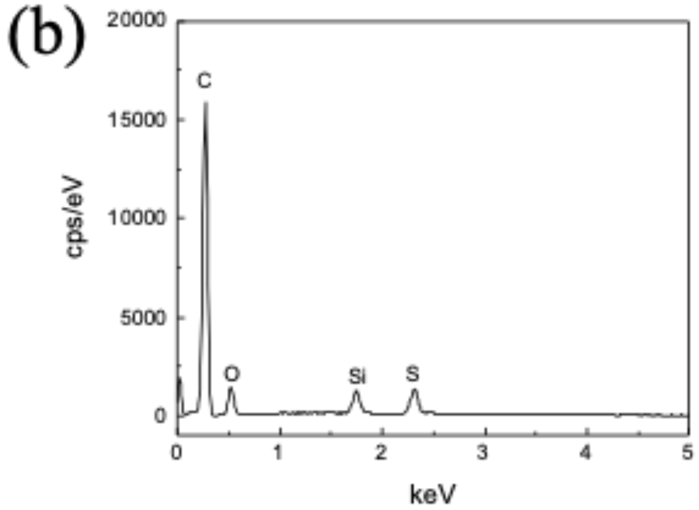

Figure S2 Energy dispersion spectroscopy (EDS) analysis of (a) a TIM and (b) a p-TIM treated with 1 wt% silane solution. The detection of Si confirms the existence of the silane molecules on the surface of the p-TIM. The EDS spectrum was measured by X-Max^N, Oxford Instruments.

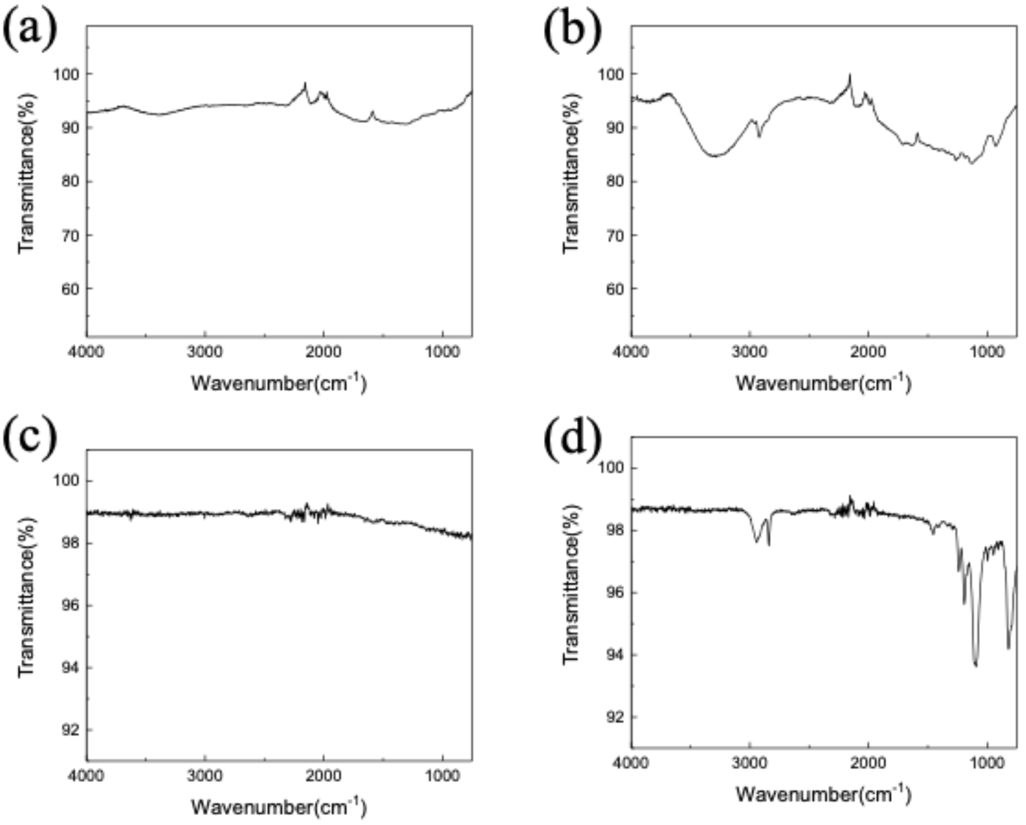

Figure S3 Fourier transform infrared (FTIR) spectra of a TIM (a) before and (b) after post-treatment with 1 wt% silane solution. FTIR spectra of a Cu surface (c) before and (d) after treatment with 1 wt% silane solution. New bands at 2920 cm^{-1} in (b) and 2838 and 2941 cm^{-1} in (d) are associated with the existence of the methylene groups from the silane coupling agent. The FTIR spectra was measured by Nicolet Continuum, Thermo Scientific.

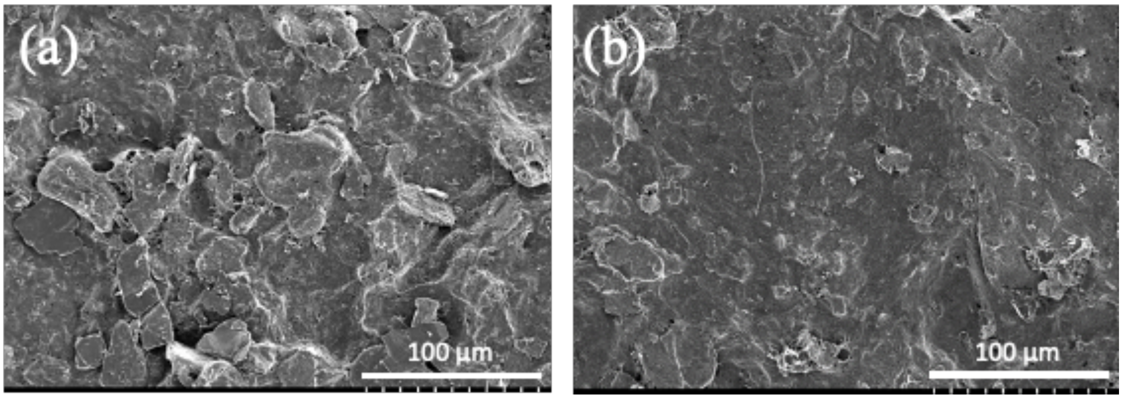

Figure S4 FE-SEM images of a TIM surface (a) before and (b) after applying 0.61 MPa of normal pressure.
